# Can Volunteer Community Health Workers Decrease Child Morbidity and Mortality in Southwestern Uganda? An Impact Evaluation

**DOI:** 10.1371/journal.pone.0027997

**Published:** 2011-12-14

**Authors:** Jennifer L. Brenner, Jerome Kabakyenga, Teddy Kyomuhangi, Kathryn A. Wotton, Carolyn Pim, Moses Ntaro, Fred Norman Bagenda, Ndaruhutse Ruzazaaza Gad, John Godel, James Kayizzi, Douglas McMillan, Edgar Mulogo, Alberto Nettel-Aguirre, Nalini Singhal

**Affiliations:** 1 Faculty of Medicine, Department of Pediatrics, University of Calgary, Calgary, Alberta, Canada; 2 Faculty of Medicine, Department of Community Health, Mbarara University of Science and Technology, Mbarara, Uganda; 3 Faculty of Medicine, Department of Community Health Sciences, University of Calgary, Calgary, Alberta, Canada; 4 Faculty of Medicine, Department of Pediatrics, University of Alberta, Edmonton, Alberta, Canada; 5 Department of Health and Education, Icelandic International Development Agency, Kampala, Uganda; 6 Faculty of Medicine, Department of Pediatrics, Dalhousie University, Halifax, Nova Scotia, Canada; Aga Khan University, Pakistan

## Abstract

**Background:**

The potential for community health workers to improve child health in sub-Saharan Africa is not well understood. Healthy Child Uganda implemented a volunteer community health worker child health promotion model in rural Uganda. An impact evaluation was conducted to assess volunteer community health workers' effect on child morbidity, mortality and to calculate volunteer retention.

**Methodology/Principal Findings:**

Two volunteer community health workers were selected, trained and promoted child health in each of 116 villages (population ∼61,000) during 2006–2009. Evaluation included a household survey of mothers at baseline and post-intervention in intervention/control areas, retrospective reviews of community health worker birth/child death reports and post-intervention focus group discussions. Retention was calculated from administrative records. Main outcomes were prevalence of recent child illness/underweight status, community health worker reports of child deaths, focus group perception of effect, and community health worker retention. After 18–36 months, 86% of trained volunteers remained active. Post-intervention surveys in intervention households revealed absolute reductions of 10.2% [95%CI (−17.7%, −2.6%)] in diarrhea prevalence and 5.8% [95%CI (−11.5%, −0.003%)] in fever/malaria; comparative decreases in control households were not statistically significant. Underweight prevalence was reduced by 5.1% [95%CI (−10.7%, 0.4%)] in intervention households. Community health worker monthly reports revealed a relative decline of 53% in child deaths (<5 years old), during the first 18 months of intervention. Focus groups credited community health workers with decreasing child deaths, improved care-seeking practices, and new income-generating opportunities.

**Conclusions/Significance:**

A low-cost child health promotion model using volunteer community health workers demonstrated decreased child morbidity, dramatic mortality trend declines and high volunteer retention. This sustainable model could be scaled-up to sub-Saharan African communities with limited resources and high child health needs.

## Introduction

Globally, 7.7 million children under the age of five die each year [1]. Almost half of all child deaths occur in sub-Saharan Africa, mostly from conditions that are preventable and treatable [2]. Delivering simple and effective child survival interventions to those needing them most remains a challenge. Uganda has approximately 190,000 child deaths each year [2] and is not on track to achieve Millennium Development Goal 4 targets [3]. Primary health care delivered by community health workers is a potential way to extend the reach of child health promotion interventions to rural communities and evidence for community health workers improving child health is mounting [4], [5], [6]. Governments and donors, including those in Uganda, are scaling up efforts to train tens of thousands of community health workers [6].

Gaps remain in the community health worker literature. More evidence of community health worker effectiveness in different settings is needed, using varied research methods [4], [5]. To date, most large community health worker studies have been from south Asia, and have assessed maternal and newborn care packages or child health programs where community health workers provide some component of curative care such as medicine distribution for pneumonia, malaria and/or diarrhea. There are few studies from Africa [4], [6] and the effectiveness of community health workers serving in a ‘health promotion-only’ role has not been well established, despite potential cost and sustainability benefits. Other priority questions include how to best integrate community health worker programs into existing government health systems [4], [6], [7], and how to retain community health workers once trained [6], [8]. Though retention is a key factor for community health worker program sustainability, relatively few published studies report retention rates, especially amongst volunteers.

Healthy Child Uganda is a Ugandan-Canadian university partnership formed to strengthen child health capacity in southwest Uganda. In response to the dire child health situation in local communities, Mbarara University of Science and Technology, local health districts and Canadian partners have developed, implemented and evaluated a child health promotion program delivered by a network of trained volunteer community health workers.

This article reports on an impact evaluation conducted using quantitative and qualitative tools to assess:

- The effect of volunteer community health workers serving in a ‘health promotion-only’ role on child morbidity, mortality and household health promoting behaviors in rural Uganda and;
- Retention of volunteer community health workers over the 3-year study period.

## Methods

### Ethics Statement

Ethics approval was obtained through the Mbarara University of Science and Technology Institutional Ethical Review Committee and the University of Calgary Conjoint Health Research Ethics Board, each of whom specifically approved this study. Informed consent was obtained prior to participation in all surveys. A written consent form was read verbatim in local dialect to each potential respondent prior to participation in the study; consent was indicated by respondents through either signature or thumbprint below the consent passage. This process for consent and the consent form were approved by both ethics committees and are consistent with survey practices used in similar settings with low literacy for scientific studies.

### Study Area and Population

Mbarara University of Science and Technology had strong established relationships in study communities in Mbarara and Bushenyi districts in southwestern Uganda. Most local ‘villages’ in the region have 300–600 people with ∼20% below five years old while ‘parishes’ comprise about 10 villages within close proximity [9], [10]. Consistent with many other Ugandan rural communities [11], local families depend mainly on subsistence farming and many live in extreme poverty. Government health centres are challenged by staffing, infrastructure and equipment shortages [12].

### Study Design

The Healthy Child Uganda volunteer community health worker model (*Figure S1*) was developed and rolled out starting in 2004. The initiative was developed as a community-based intervention project rather than a research study, though the intent was to document outcomes and change from baseline where possible.

**Figure 1:**
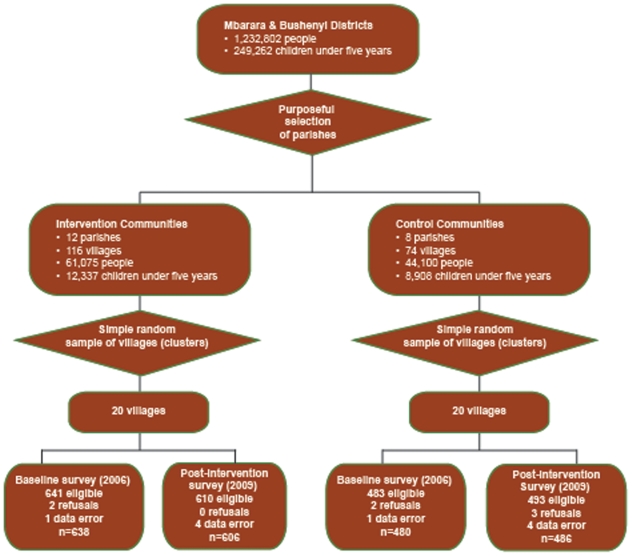
Household survey study design. Algorithmic illustration of methodology and sample size for both intervention and control areas in household survey. Population data from Mbarara and Bushenyi Districts, 2008 estimates [9], [10]. Data errors noted in figure were due to missing or uninterpretable ages. In addition, there were 9 surveys conducted in 2009 that were missing village identification so are not shown. All surveys with data errors were excluded from analysis.

In this paper, we report the impact on communities who received this intervention between 2006 and 2009 based on multiple evaluation tools. Data collection methods included: (A) a household survey with baseline and post-intervention comparisons of intervention and control populations (*Figure 1*); (B) retrospective review of a community health worker registry; (C) retrospective review of community health workers' monthly birth and child death reports; and (D) focus group discussions.

In 2005, prior to intervention, local government-appointed health centre management teams purposefully chose 12 parishes (4 parishes in each of 3 geographically separate health sub-districts representing approximately 5% of the total population of these areas) to receive the community health worker intervention (*Figure 1*). Selection of intervention communities was based on high child health needs (i.e. difficult access to healthcare services, perceived high incidence of illness, malnutrition and child deaths) as perceived by management teams and was not randomized. Local investigators also identified 8 parishes in the same health sub-districts to serve as controls for evaluation purposes. They were nearby in location, served by the same sub-district health centres, thought to have similar geography, cultural and religious representation, and were not randomized.

Intervention communities (total population ∼61,000; under five years ∼12,300) received child health promotion provided by trained community health workers while control communities received the usual government and non-governmental health services only. Usual government health services at the time of study included mainly primary and secondary health services through local health centres staffed by health professionals such as nurses, midwives and clinical officers but rarely medical doctors. Outreach visits by government health workers were uncommon. Care seeking from traditional providers including traditional birth attendants and healers was common. No training of child health community health workers or consistent program for community health workers was present prior to the project in either intervention or control areas, or in control areas throughout the study period.

#### A. Household Survey

A household survey, based on the Knowledge, Practices, and Coverage Survey (KPC) 2000+ Rapid Catch [13] was conducted in 2006 (baseline) and repeated in 2009 (post-intervention). Questionnaires were constructed in English, translated into local dialect, and back translated. Following a field pilot in 3 villages, minor translation, rewording and question order changes were made to about 10% of questions for clarity.

The number of villages for the household survey, determined based on budget feasibility, was 39: twenty intervention villages and nineteen control villages (clusters). To select villages for household survey, the names of all intervention villages were typed into SPSS. Using the SPSS randomization function, a minimum of 1 and a maximum of 2 villages were identified from each parish for survey. The process was repeated for control areas until a minimum of 2 and a maximum of 3 villages per parish were chosen.

Trained research assistants, who had not been involved in the intervention, administered surveys verbally. According to KPC 2000^+^ Rapid Catch methodology [13] all households in the selected survey villages were considered eligible if one or more children under two years of age lived there with their biological mother who was available for survey. The same selected villages were visited during both the baseline and post-intervention survey. Questionnaires asked eligible mothers about attitudes, personal and household health-related practices and health of the youngest child. The process for identifying eligible homes was the same for both surveys.

#### B. Community Health Worker Database

The Healthy Child Uganda project office established and maintained an MS Excel database containing names, sex, village and training date for all trained community health workers. The database was updated quarterly to reflect any ‘finish dates’ for community health workers who died or were replaced and add details about community health worker replacements. Additional demographic data was collected periodically including date of birth and highest education level achieved.

#### C. Birth and Child Death Reports

Since reliable vital registration was unavailable, community health workers were asked to report live births and deaths in children under five years old in their villages monthly. Reports were only available from intervention communities since no community health workers were trained in control areas. Written community health worker reports from intervention areas were collected and collated monthly by health centre supervisors, then submitted to the Healthy Child Uganda office.

#### D. Focus Group Discussions

Conducted in 2009, each focus group involved 7–9 individuals of the same gender from randomly selected intervention villages. Focus group discussions were not conducted in control areas. Recruitment was purposeful involving parents who were identified by local council leaders and research assistants and who were available and willing to participate. Local research assistants who had not been involved in Healthy Child Uganda presented a series of open-ended questions in the local language about intervention impact and answers were recorded on audiocassettes, transcribed and translated into English.

### Intervention

During 2006–2009, Healthy Child Uganda trained volunteer community health workers and supported their child health promotion activities in intervention communities. There were no trained child health community health workers in control communities.

Mbarara University of Science and Technology staff and faculty developed curricula, managed field activities and coordinated evaluation. With technical support from district health services and Canadian partners, university staff and faculty also conducted community health worker training. Health sub-district medical managers selected local health centre staff representing a variety of health backgrounds (i.e. midwives, nurses) as ‘trainers’ to train and supervise the volunteer community health workers. All trainers attended 15 or more days of training in curriculum content and leadership, conducted by Mbarara University and Canadian faculty.

Community health worker selection meetings were held in each village, attended by trainers who outlined intervention goals and community health worker expectations and responsibilities. In each village, community members identified their own criteria for selection such as desirable personal qualities, experience, education level, marital status, age, etc. Each village then determined a process and selected two volunteer community health workers. Selected volunteers attended an initial 5-day course, held within the communities. A participatory curriculum stressed Community-IMCI guidelines [14], key family care practices (*Table S1*), problem-solving and basic child assessment skills. While emphasis was placed on health promotion (i.e good nutrition, immunization, prenatal care, safe delivery and disease-specific prevention such as bed net use), early care seeking for illness was also underscored and identification of danger signs in young children and pregnant women were taught. Simple home treatments of illness such as use of oral rehydration salts for diarrhea were also imparted.

**Figure 2:**
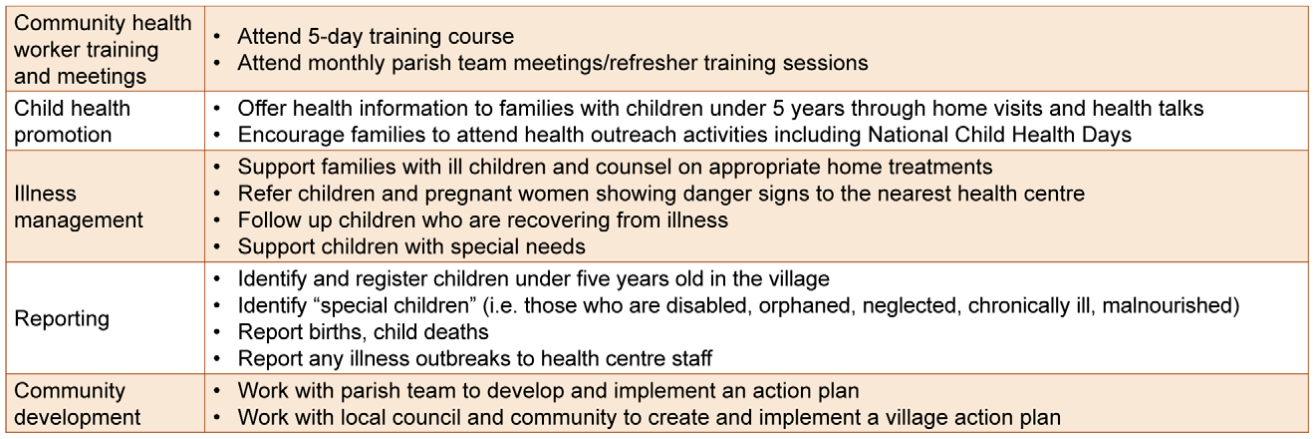
Volunteer community health worker responsibilities.

Volunteer community health workers were organized into parish teams for initial training and for monthly meetings with trainers held throughout the study period. Monthly meetings included 2-hour refresher training sessions and presentation of village reports. Each community health worker received only a nominal transport stipend (∼$1.50USD) per meeting or training day. Non-financial incentives included t-shirts (one each), certificates (following initial training and annual), exchange visits between parish teams (once in three years), inter-village competitions (variable frequency), annual ‘holiday’ gift (valued at about $3USD) and income-generating project training for parish teams (ad hoc). Main tasks and responsibilities of volunteer community health workers are outlined in *Figure 2*. Community health workers served on average, 45 children under age five years each, living in 25 households.

If a volunteer community health worker died or resigned, a replacement was selected and was trained as soon as feasible. Initial training courses for replacement community health workers were conducted once or more per year.

A one-time distribution of an insecticide-treated mosquito net (between October 2007 and June 2008) to each intervention home with a young child and/or pregnant woman was made possible by donation from an outside organization. There was no similar distribution within control communities, however, during the intervention period, the Ugandan government and non-governmental organizations did organize free and for-cost distribution in some local communities.

During the study period, Healthy Child Uganda also facilitated minor physical upgrades at local health centres and provided World Health Organization-based Integrated Management of Childhood Illness (IMCI) training [15] for local health providers. Most of the health centres where such upgrades and training occurred served both intervention and control communities.

More details of the Healthy Child Uganda volunteer community health worker model can be found at www.healthychilduganda.org/resources.

### Data Analysis

All individuals who conducted data analysis were not involved in the intervention.

Household survey data was analyzed using the ‘R’ statistical package [16]. Descriptive statistics for community health worker demographics comprised the use of means and range for numerical data and proportions for categorical data. Regarding outcomes, indicators were calculated for intervention and control villages for each survey (2006 and 2009). These indicators are dichotomous type of data, therefore proportions were calculated; confidence intervals were calculated for the difference in proportions between 2006 and 2009 within each of the intervention and control groups, adjusting for the clustered nature of the data (cluster effects). The intra-class correlation coefficient and the corresponding design effects were based on the data and cluster structure at baseline for each indicator (*Figure S2*).

Births and child deaths for each parish were retrospectively reviewed. Complete data for a consecutive 18-month period (but not a full 24 months) were available for most parishes, so data were grouped into biannual (6 month) sequential time intervals starting immediately following completion of initial community health worker training. Parishes with fewer than 18 months of consecutive data were excluded (note that initial training by parish was staggered). Proportions of births and deaths for each six month interval were determined by dividing the total absolute deaths/births per interval by the estimated under-5 year old parish population [9], [10].

For focus groups, two researchers separately conducted a primary coding analysis determining main themes and sub-themes of the transcripts. Researchers shared findings and attained a consensus to label prominent themes. To determine inter-rater reliability of allocation of these primary themes, a kappa value [17] was calculated.

## Results

### Coverage and community health worker demographics

Volunteer community health workers were trained in all 118 intervention villages in the 12 intervention parishes. Based on village population data, 61,075 people including 12,337 children under age five years were living in communities served by HCU-trained community health workers. Including original and replacement community health workers, a total of 271 volunteers were trained (64.8% female) during the intervention period.

In December 2009, there were 201 volunteers for whom demographic data could be confirmed. Within this cohort, mean age was 34.9 years (range 20–73); 35% had attended school but had not completed primary school (P7) while 65% had completed primary school, half of whom were educated beyond P7.

### Community health worker retention

By July 2009 (18–36 months following initial community health worker training owing to staggered start dates), 234 (86.3%) of all trained community health workers (original and replacement) were still active. According to transport refund registers, monthly meeting attendance and reporting exceeded 95% of active volunteers.

### Household surveys

At baseline (2006), 1,124 households were identified as eligible for survey. Four households refused to participate (2 intervention, 2 control) and 2 surveys were missing child age data (1 in intervention and 1 in control); thus, 1,118 baseline surveys were analyzed (638 intervention, 480 control). Post-intervention (2009), 1,112 households were deemed eligible. There were 3 refusals (all control). Seventeen surveys were missing either village name (9) or child age (8); these were excluded leaving 1,092 surveys for analysis (606 intervention, 486 control). Intervention/control and baseline/post-intervention comparisons revealed no differences with respect to gender/age of the youngest child or maternal age (*Table 1*). A lower schooling level was noted in both surveys in intervention mothers compared with controls.

**Table 1:** Household characteristics at baseline (2006) and post-intervention (2009) in intervention and control communities.

	2006-Baseline		2009-Post Intervention	
	Intervention (n = 638)	Control (n = 480)	Intervention (n = 606)	Control (n = 486)
**Household**				
Median Number of Children <5 Years	2	2	2	2
Median Number of People in Household	5	5	6	5
**Mother**				
Mean Maternal Age (years)	27.0	26.4	27.1	26.3
Median Maternal Highest Level of Education* (%)				
*None*	27	22	24	14
*P1–P3*	21	16	16	14
*P4–P7*	46	50	52	54
*S1 and above*	7	13	8	19
**Youngest Child**				
Mean Age (months)	11.0	10.6	10.5	10.1
% Male	49	52	51	50

P = Primary; Primary school includes P1–P7 and is followed by secondary school (S).

In intervention areas, 24% [95% C.I. 17.8, 31.0] reported having had a community health worker visit their home within the past month; 22% [95% C.I. 17.1, 26.7] reported a community health worker had assessed their youngest child during illness.

In intervention villages there was a statistically significant reduction in the combined prevalence of the three most common illnesses, and of diarrhea specifically; these reductions apparently were greater in intervention than in control communities (*Table 2* *, Table S2*). Clinically significant reductions in prevalence of under nutrition and fever/malaria were also seen. Three health promotion indicators illustrating main intervention targets showed improvements (*Table 2* *, Table S2*): mosquito net use and measles immunization were significantly increased and attendance at antenatal care also improved following the intervention.

**Table 2:** Nutritional status, recent illness, health promoting behaviors; intervention/control groups, at baseline and post-intervention.

Indicator	Baseline (2006)		Post-intervention (2009)		Absolute Change: 2009–2006 (95% CI)^1^			
	Intervention	Control	Intervention	Control	Intervention		Control	
**Nutritional Status, <24 months old, as assessed by research assistant**								
Underweight^2^	18.3%	13.8%	13.2%	12.8%	−5.1%	(−10.7%, 0.4%)	−1.0%	(−6.9%, 4.9%)
**Recent illness, as reported by mother, past two weeks, in youngest child <24 months old**								
Any of fever/malaria, diarrhea, fast/difficult breathing^3^	62.2%	55.8%	51.2%	53.7%	−11.1%	(−18.5%, −3.6%)	−2.1%	(−10.7%, 6.4%)
Fever/malaria^4^	34.2%	29.4%	28.5%	28.0%	−5.8%	(−11.5%, −0.003%)	−1.3%	(−7.7%, 5.0%)
Diarrhea^5^	45.0%	39.0%	34.8%	36.2%	−10.2%	(−17.7%, −2.6%)	−2.8%	(−11.3%, 5.7%)
Fast/difficult breathing^6^	15.8%	14.6%	14.1%	17.3%	−1.8%	(−6.9%, 3.4%)	2.7%	(−3.3%, 8.7%)
**Health promoting behaviours**								
Mosquito net seen in home	10.2%	19.0%	47.4%	31.5%	37.2%	(28.6%, 45.7%)	12.5%	(2.5%, 22.6%)
Measles vaccine^7^	61.7%	60.4%	72.3%	67.2%	10.6%	(1.6%, 19.7%)	6.8%	(−4.1%, 17.7%)
Antenatal care attendance 4 or more times during last pregnancy	33.9%	45.8%	40.8%	52.5%	6.9%	(−2.4%, 16.2)	6.6%	(−4.2%, 17.5%)

CI = confidence interval, ITN = insecticide-treated net. 2006: n = 1118 (638 intervention, 480 control); 2009: n = 1092 respondents (606 intervention, 486 control).

1 CI shown are adjusted for cluster effect for each indicator, based on baseline data.

2 z-score greater than 2 standard deviations below median weight-for-age, according to 2006 WHO growth standards [26].

3 missing response: 1 in control (2006),

4 missing responses: 1 in intervention (2006), 2 in intervention (2009), 1 in control (2009),

5 missing responses: 1 in intervention (2009), 2 in control (2009),

6 missing data: 2 in intervention (2009), 1 in control (2009),

7 By maternal report, in children 12–23 months.

### Child deaths

Live birth and child death (under five years old) data from 10 of 12 intervention parishes is shown (*Table 3*) .The two excluded parishes had not yet completed 18 months post training at time of analysis. Birth/death data were not available from control parishes. The proportion of child deaths for all intervention parishes combined, decreased from 1.54 in interval 1, to 0.72 in interval 3 which was a relative decline of 53.2%. During the same period, the overall proportion of births across parishes showed a relative decline of 28.2%, which was significant. A household audit of child deaths was conducted using research assistants who travelled door-to-door in 24 randomly selected intervention villages. By comparison with household reporting of deaths, community health workers had reported 18% overall fewer child deaths during one calendar year.

**Table 3:** Child deaths during first 18 months of intervention, per parish and time interval, by community health worker report.

			Absolute number of deaths in children under five years old (rate)^1^					
Parish	Under 5 year old population (2008)^2^	Reporting start date (yr-mm)	Interval 1	(intervention start to 6 months post)	Interval 2	(7–12 months post intervention start)	Interval 3	(13–18 months post intervention start)
A	1555	06-Jul	43	(2.76)	34	(2.19)	25	(1.61)
B	1030	07-Mar	16	(1.55)	20	(1.94)	6	(0.58)
C	1394	06-Jul	12	(0.86)	14	(1.00)	8	(0.57)
D	808	07-Mar	12	(1.48)	7	(0.87)	7	(0.87)
E	929	07-Mar	19	(2.04)	5	(0.54)	5	(0.54)
F	662	07-Apr	6	(0.91)	0	(0.00)	2	(0.30)
G	1071	06-Jul	14	(1.31)	6	(0.56)	9	(0.84)
H	970	07-Sep	10	(1.03)	12	(1.24)	5	(0.52)
I	505	07-Aug	5	(0.99)	4	(0.79)	5	(0.99)
J	1151	07-Aug	28	(2.43)	5	(0.43)	4	(0.35)
**All Parishes**	**10075**		**217**		**136**		**108**	
**Mean proportion** 1 **all parishes (95% CI)**			**1.54**	**(1.33–1.65)**	**0.96**	**(0.75–1.17)**	**0.72**	**(0.60–0.84)**

CI = confidence interval. Data are available for intervention parishes only. Reporting start date varies with staggered training and intervention start dates. Intervals represent the time elapsed post intervention start in each parish. Data from 10/12 intervention parishes presented here; remaining 2 parishes did not have 18 months of data available at time of analysis due to staggered intervention start date.

1 Proportion of child deaths is calculated as absolute under five year old deaths per under 5 year old population;

2 Population estimates for 2008, Mbarara and Bushenyi Districts [9], [10] represents nearest to mid-intervention estimates available.

### Focus group discussions

Main focus group themes are shown (*Table 4*). The calculated Kappa value, representing inter-rater reliability, was 0.88. Most participants felt the volunteer community health workers had direct and positive effects on child health and survival, expressed gratitude for the education they provided, and expressed confidence in their own ability to improve child health and save their children's lives. Volunteer community health workers were credited with improved home, community and care-seeking practices. Participants attributed certain changing gender roles and community development to the intervention including new business opportunities, improved pregnancy/childbirth practices, improved home environments and personal hygiene, and improved status of women within their families and villages.

**Table 4:** Post-intervention focus group discussion main themes and exemplary quotations.

Theme	Exemplary Quotation
Decreased child mortality	“Cleanliness around homesteads has decreased the deaths among children. They used to share plates, cups, and beds when one child was sick and another wasn't, and they never used to wash their hands after using the latrine. This could spread diarrhea, which was a major sickness in our children. Now, since we got taught about how to deal with these things, deaths have decreased …”
Improved use of primary medical care	“‘Community health workers’ have brought medicines, like de-worming tablets, called us for polio vaccinations, and made sure that we went. They have advised us on the status of our children, like if they are underweight, malnourished, and so on. Our children are quickly attended to because the ‘community health workers’ give you a small note when you go to the health centre, since they have a good working relationship with them.”
Increased knowledge of disease prevention	“There is change, in that parents now know about how to feed their children properly, and about hygiene, hence eliminating disease.”
Improved child feeding and nutrition	“We know how to feed our children appropriately with the right foods. We know how often to feed them. We did not know before– we were ignorant of this information.”
Enhanced parent-child bonding	“I give my child time with me. I study his ways, how he laughs, eats, if he feels weak or energetic. We have a special bond. I love him more due to the good things I see in him and I love watching him grow.”
Facilitated community development	“You find that everyone tries to get involved in sanitation around their homes. Now, if you find that each home has fruits in the garden and safe, protected drinking water, that they sleep well and have mosquito nets, then you won't have children who fall sick all the time. You find yourself saving this money and using it for the development of your home and you are able to put children in school.”

Inter-rater agreement: Kappa = 0.88.

## Discussion

A community-based intervention using volunteer community health workers to promote health for children under five years old in rural Uganda successfully improved child health outcomes and decreased mortality. Household survey findings of reduced malnutrition and morbidity (from household surveys compared to controls and from focus group reports) were complemented by evidence of improved child health practices and reductions in reported deaths (from focus group reports and from community health worker monthly death reports), suggesting that health improvements went beyond illness reduction, perhaps dramatically decreasing the mortality rate within a short time.

These results support other recent studies which have reported improved newborn and child survival following community health worker programming, including several controlled intervention studies in south Asia [18], [19], [20], [21]. Our study is complementary to global studies and provides new and important evidence of success of improved under five-year-old child health outcomes in sub-Saharan Africa, using community health workers in a health promotion-only role and within a government-integrated program. Reductions in child mortality and disease burden in intervention groups may reflect improved care-seeking practices, sanitation, hygiene, insecticide-treated mosquito net use and nutrition resulting from a range of community health worker-related activities including, but not limited to, parental health education, insecticide-treated mosquito net distribution, and active volunteer and community engagement. A participatory approach and solid volunteer support may have encouraged impact beyond direct health outcomes to broader determinants of health like gender and economic opportunities, as also described during focus group discussions. Specific features of the Healthy Child Uganda approach that promote success may have included: attention to local needs and priorities, alignment with local health systems, an established and consistent selection process for community health workers, training appropriate to the setting, and regular supervision; all of these have been identified as important contributors to other successful community health worker programs [4], [5], [6], [7], [22].

During the three years of the intervention, volunteer community health worker retention was notably high at 86%. This is especially remarkable since volunteer-based community health worker programs have generally been associated with lower retention [23]. Several recent studies have reported quite low retention of both volunteers and non-volunteers; 52–70% after 36 months amongst paid community health workers in Bangladesh [24] and 33% after 11 months amongst volunteer AIDS-support community health workers in Kenya [25]. Healthy Child Uganda experience suggests that within our setting, high retention of volunteer community health workers may be possible. We speculate that our model may facilitate volunteer retention through the careful selection process, community choice of volunteers, clear volunteer expectations, and ongoing, participatory training in teams with supportive supervision and regular meetings. We recognize that our high retention over the short to medium term may represent enthusiasm and commitment which could be harder to sustain over time in a volunteer system—further studies to better understand how to motivate community health workers long-term will be critical.

This evaluation demonstrates that through combining operational data with qualitative and quantitative surveys, a meaningful assessment of a real world model in action can be realized. Use of mixed qualitative and quantitative data for evaluation may be a feasible option for similar community-based programs where research funding is limited, communities are remote, and vital registration is lacking.

Our ability to fully interpret morbidity and household behaviour evaluation findings is limited both by study design challenges and to bias inherent in pre/post study design. Randomization at the intervention unit level (parishes) was lacking. Also, due to geographic proximity, contamination in control communities is possible; however, contamination in control sites would have washed out the difference between the arms if there had been any effect due to contamination. As well, using disease prevalence estimates from only two data points (i.e. pre and post intervention) and use of maternal report for data collection could ignore other potential factors contributing to disease burden and recall bias. However, use of the same survey tool during the same months of the year for both pre and post household surveys and use of a control group for comparison does strengthen interpretation. Statistically significant improvements in intervention areas where none exist in controls, supported by focus group findings certainly suggests intervention impact though absolute burden of illness indicators may be less accurate, especially since baseline burden of illness indicators for control sites were consistently lower than in intervention areas. Finally, community health workers were encouraged to choose their own focus for health promotion activities from a broad menu, which may have lessened impact on selected reporting indicators. However, such program flexibility alternatively may have contributed to better targeting and overall child health and volunteer retention.

Mortality was not an indicator we had anticipated reporting at intervention start. However, given available operational death data and anecdotal evidence of decreased deaths, retrospectively analyzing monthly community health worker death reports, as part of this evaluation seemed reasonable and important. A lack of available birth and death data from control communities and audit findings suggesting potential community health worker under-reporting of births and deaths makes mortality impact interpretation difficult. Interpretation is also complicated by substantial reduction in births following community health worker introduction. Whether reports of declining births resulted from increased female empowerment, education of women around contraception and birth spacing, reduced reporting, or a combination of factors, is not clear. However, a consistent reporting system was used throughout the intervention period without incentives for over/underreporting, the decrease in reported child mortality was dramatic, and field, health worker, and focus group reports corroborate this decline, suggesting a real and important trend in improved child survival.

Further research questions remain to be answered: Can child health improvements be sustained and volunteers be retained over 5/10 years? Can the program be modified and/or replicated and scaled up? Would increasing community health worker practice scope to include curative care change retention or further impact child health outcomes? Can volunteer community health workers be used to effectively track births and deaths in regions where no vital registries exist?

The Healthy Child Uganda model has ingredients favorable for scaling up—it is community-driven and locally run, low-cost, uses local resources, and is well integrated with the government health system. Major study findings of high volunteer retention and measurable and rapid child health improvements support the potential for an affordable and sustainable volunteer community health worker cadre which is good news in Uganda where a national community health worker program (Village Health Teams [12]) is currently being rolled out. However, for such national or regional programs to succeed, the importance of community engagement and community health worker selection, training, and supervision cannot be underestimated or compromised. Programs must be flexible to accommodate modifications based upon ongoing critical evaluations, local needs and resources. The Healthy Child Uganda volunteer community health worker model demonstrates one compelling way to improve child survival in sub-Saharan Africa and may serve as a successful example for other communities with similar limited health and healthcare provider resources and high child health needs.

## Supporting Information

Figure S1 **Key Elements of the Healthy Child Uganda Volunteer Community Health Worker Model.** A pictoral representation.
(TIF)

Figure S2 **Statistical Formulas Used.** Describes formulas for Design Effect and 95% Confidence Interval calcuations, from Donner A, Birkett N, Buck C (1981) Randomization by cluster. Sample size requirements and analysis. Am J Epidemiol 114: 906–914.
(TIF)

Table S1 **IMCI key family practices emphasized by Healthy Child Uganda volunteer community health workers.** Adapted from WHO, 2009; UNICEF. http://www.unicef.org/health/index_imcd.html. Access Date: April 1, 2011.
(DOC)

Table S2 **Nutritional status, recent illness, health promoting behaviors.** Intervention/control groups, at baseline and post-intervention including design effect and absolute value details. CI = confidence interval, Num = numerator; Den = denominator; Prop = proportion; ITN = insecticide-treated net. 2006: n = 1118 (638 intervention, 480 control); 2009: n = 1092 respondents (606 intervention, 486 control).1 CI shown are adjusted for cluster effect for each indicator, based on baseline data. 2 z-score greater than 2 standard deviations below median weight-for-age, according to 2006 WHO growth standards [26]. 3 missing response: 1 in control (2006), 4missing responses: 1 in intervention (2006), 2 in intervention (2009), 1 in control (2009), 5missing responses: 1 in intervention (2009), 2 in control (2009), 6missing data: 2 in intervention (2009), 1 in control (2009), 7By maternal report, in children 12–23 months.
(DOC)
