# Supplementary figures and images for: Can Volunteer Community Health Workers Decrease Child Morbidity and Mortality in Southwestern Uganda? An Impact Evaluation
Source: PLoS One. 2011 Dec 14;6(12):e27997. doi: 10.1371/journal.pone.0027997 (PMC3237430; doi:10.1371/journal.pone.0027997)

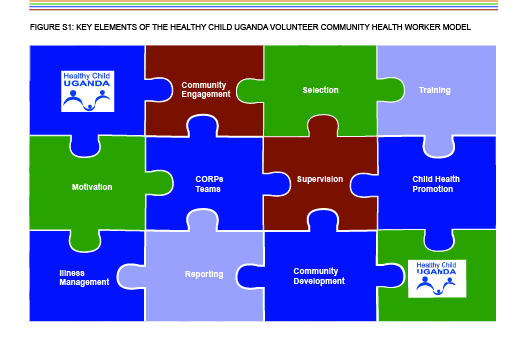

Supplement: Figure S1 — Key Elements of the Healthy Child Uganda Volunteer Community Health Worker Model. A pictoral representation. (TIF) [file pone.0027997.s001.tif]

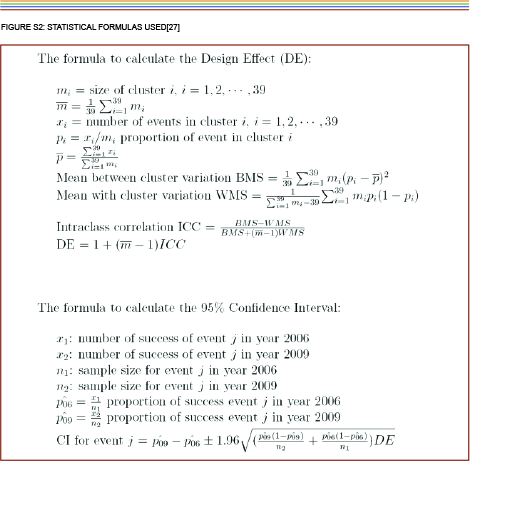

Supplement: Figure S2 — Statistical Formulas Used. Describes formulas for Design Effect and 95% Confidence Interval calcuations, from Donner A, Birkett N, Buck C (1981) Randomization by cluster. Sample size requirements and analysis. Am J Epidemiol 114: 906–914. (TIF) [file pone.0027997.s002.tif]
