# Supplementary material for: Can Volunteer Community Health Workers Decrease Child Morbidity and Mortality in Southwestern Uganda? An Impact Evaluation
Source: PLoS One. 2011 Dec 14;6(12):e27997. doi: 10.1371/journal.pone.0027997 (PMC3237430; doi:10.1371/journal.pone.0027997)
Supplement: Table S1 — IMCI key family practices emphasized by Healthy Child Uganda volunteer community health workers. Adapted from WHO, 2009; UNICEF. http://www.unicef.org/health/index_imcd.html. Access Date: April 1, 2011. (DOC) [file pone.0027997.s003.doc]

**Table S1: IMCI key family practices emphasized by Healthy Child Uganda volunteer community health workers adapted from WHO, 2009**

| Promoting a child’s growth and development | • Breastfeed babies exclusively for six months  • From six months, give children quality complementary foods, while continuing to breastfeed for two years or longer  • Ensure that children receive enough micronutrients — such as vitamin A, iron and zinc — in their diet or with supplements  • Stimulate mental and social development by responding to a child’s needs for care, and by playing, talking and providing an interesting environment |
| --- | --- |
| Disease prevention | • Dispose of all feces safely, wash hands after defecation, before preparing meals, and before feeding children  • Protect children in malaria-endemic areas, by ensuring that they sleep under insecticide-treated bed nets  • Provide appropriate care for people affected by HIV/AIDS, especially orphans, and take action to prevent the spread of HIV infection |
| Appropriate care at home | • Continue to feed and offer more fluids, including breast milk, to children when they are sick  • Give sick children appropriate home treatment for infections  • Protect children from injury and provide treatment for minor cuts and scrapes  • Prevent child abuse and neglect, and take action when it does occur  • Involve fathers in the care of children and in family reproductive health |
| Care-seeking outside the home | • Recognize when a sick child needs treatment outside the home, and seek care from appropriate providers  • Take children to complete a full course of immunization before their first birthday  • Follow the health care provider’s advice on treatment, follow-up and referral  • Ensure that every pregnant woman has adequate prenatal care, and that she seeks appropriate care for her delivery and afterward |
