# Supplementary material for: Can Volunteer Community Health Workers Decrease Child Morbidity and Mortality in Southwestern Uganda? An Impact Evaluation
Source: PLoS One. 2011 Dec 14;6(12):e27997. doi: 10.1371/journal.pone.0027997 (PMC3237430; doi:10.1371/journal.pone.0027997)
Supplement: Table S2 — Nutritional status, recent illness, health promoting behaviors. Intervention/control groups, at baseline and post-intervention including design effect and absolute value details. CI = confidence interval, Num = numerator; Den = denominator; Prop = proportion; ITN = insecticide-treated net. 2006: n = 1118 (638 intervention, 480 control); 2009: n = 1092 respondents (606 intervention, 486 control).1 CI shown are adjusted for cluster effect for each indicator, based on baseline data. 2 z-score greater than 2 standard deviations below median weight-for-age, according to 2006 WHO growth standards [26]. 3 missing response: 1 in control (2006), 4missing responses: 1 in intervention (2006), 2 in intervention (2009), 1 in control (2009), 5missing responses: 1 in intervention (2009), 2 in control (2009), 6missing data: 2 in intervention (2009), 1 in control (2009), 7By maternal report, in children 12–23 months. (DOC) [file pone.0027997.s004.doc]

**Table S2: Nutritional status, recent illness, health promoting behaviors; intervention/control groups, at baseline and post-intervention** including design effect and absolute value details

| **Indicator** | **Design Effect** | **Baseline (2006)** | | | | | | | **Post-intervention (2009)** | | | | | | **Absolute Change:** | |
| --- | --- | --- | --- | --- | --- | --- | --- | --- | --- | --- | --- | --- | --- | --- | --- | --- |
|  |  |  |  |  |  |  |  |  |  |  |  |  |  |  | **2009-2006 (95% CI)** | |
|  |  | **Intervention** | | | | **Control** | | | **Intervention** | | | **Control** | | | **Intervention** | **Control** |
|  |  | **Num.** | | **Den.** | **Prop** | **Num.** | **Den.** | **Prop** | **Num.** | **Den.** | **Prop** | **Num.** | **Den.** | **Prop** |  |  |
| **Nutritional Status, <24 months old, as assessed by research assistant** | | | | | | | | | | | | | | | | |
| Underweight ^2^ | 1.9027 | | 117 | 638 | 18.34% | 66 | 480 | 13.75% | 80 | 606 | 13.20% | 62 | 486 | 12.76% | -5.1% (-10.7%, 0.4%) | -1.0% (-6.9%, 4.9%) |
| **Recent illness, as reported by mother, past two weeks, in youngest child <24 months old** | | | | | | | | | | | | | | | | |
| Any of fever/malaria, diarrhea, fast/ difficult breathing^3^ | 1.8557 | | 397 | 638 | 62.23% | 268 | 480 | 55.83% | 310 | 606 | 51.16% | 261 | 486 | 53.70% | -11.1% (-18.5%, -3.6%) | -2.1% (-10.7%, 6.4%) |
| Fever/malaria^4^ | 1.2309 | | 218 | 637 | 34.22% | 141 | 480 | 29.38% | 172 | 604 | 28.48% | 136 | 485 | 28.04% | -5.8% (-11.5%, -0.003%) | -1.3% (-7.7%, 5.0%) |
| Diarrhea^5^ | 1.9348 | | 287 | 638 | 44.98% | 187 | 480 | 38.96% | 210 | 603 | 34.83% | 175 | 484 | 36.16% | -10.2% (-17.7%, -2.6%) | -2.8% (-11.3%, 5.7%) |
| Fast/ difficult breathing^6^ | 1.7013 | | 101 | 638 | 15.83% | 70 | 479 | 14.61% | 85 | 604 | 14.07% | 84 | 485 | 17.32% | -1.8% (-6.9%, 3.4%) | 2.7% (-3.3%, 8.7%) |
| **Health promoting behaviours** | | | | | | | | | | | | | | | | |
| Mosquito net seen in home | 3.4174 | | 65 | 638 | 10.19% | 91 | 480 | 18.96% | 287 | 606 | 47.36% | 153 | 486 | 31.48% | 37.2% (28.6%, 45.7%) | 12.5% (2.5%, 22.6%) |
| Measles vaccine^7^ | 1.3562 | | 185 | 300 | 61.67% | 128 | 212 | 60.38% | 185 | 256 | 72.27% | 129 | 192 | 67.19% | 10.6% (1.6%, 19.7%) | 6.8% (-4.1%, 17.7%) |
| Antenatal care attendance 4 or more times during last pregnancy | 2.9733 | | 216 | 638 | 33.86% | 220 | 480 | 45.83% | 247 | 606 | 40.76% | 255 | 486 | 52.47% | 6.9% (-2.4%, 16.2) | 6.6% (-4.2%, 17.5%) |
